# Exploring Basic Psychological Need Satisfaction and Relationship Quality as Protective Factors of Mental Well-Being During the COVID-19 Pandemic

**DOI:** 10.5964/ejop.13741

**Published:** 2025-02-28

**Authors:** Barbara Horvát, Tamás Martos, Claudia Chiarolanza, Viola Sallay, Ashley K. Randall

**Affiliations:** 1 Department of Personality, Clinical and Health Psychology, University of Szeged, Szeged, Hungary; 2 Department of Dynamic and Clinical Psychology, and Health Studies, Sapienza - University of Rome, Rome, Italy; 3 School of Counseling and Counseling Psychology, Arizona State University, Tempe, AZ, USA; Victoria University of Wellington, Wellington, New Zealand

**Keywords:** COVID-19 pandemic, mental well-being, basic psychological needs, relationship quality, autonomy, competence, relatedness

## Abstract

COVID-19’s global impact on mental health has been profound. To better understand factors that mitigate effects of stress, particularly during quarantine periods, this study examined roles of basic psychological need satisfaction and relationship quality in mental well-being in the context of COVID-19-related stress. Conducted from March to May 2020, this online questionnaire research involved 805 individuals in romantic relationships (mean age = 37.88 ± 12.50 years; 70.19% female). Path analysis revealed that higher mental well-being was associated with satisfaction of basic psychological needs, positive relationship quality, and lower COVID-19-related stress. Higher autonomy satisfaction was linked to lower COVID-19-related stress, whereas increased relatedness satisfaction and better relationship quality predicted higher COVID-19-related stress. The findings implicate complex associations among basic psychological need satisfaction, relationship quality, and mental well-being. While better relationship experiences might even heighten perceived stress during a global crisis, they simultaneously function as protective factors for overall mental health.

The coronavirus disease (COVID-19) emerged in 2019, and by March 2020, the World Health Organization had officially declared it a pandemic. Worldwide, people’s lives have undergone profound changes, with social distancing, quarantine measures, and lifestyle adjustments becoming the norm (e.g., Brooks et al., 2020; Wang et al., 2020.). As nations struggled to contain and prevent infections, implementation of measures such as social distancing, cancellation of public events, and quarantine became imperative (Public Health England, 2020). Although necessary to prevent the disease’s spread, isolation and quarantine adversely affected mental health (Brooks et al., 2020; Wang et al., 2020), leading to feelings of helplessness, fear, and disappointment (Taylor & Asmundson, 2021).

Results of the COVID-19 advisory initiative conducted among university members in Hungary from March 4 to May 25, 2020, show that fear and anxiety were widespread among participants, who routinely battled a variety of challenges, including persistent feelings of depression, fatigue, disrupted eating and sleeping patterns, hopelessness, and decreased interest and motivation (Szlamka et al., 2021). Despite these documented difficulties, data on interrelated aspects of mental health, fulfillment of basic psychological needs, and dynamics of interpersonal relationships caused by the COVID-19 pandemic still need to be comprehensively supplemented.

According to self-determination theory (SDT; Ryan & Deci, 2017), satisfaction of basic psychological needs (BPN) is essential for well-being (Ryan & Deci, 2017). Moreover, SDT defines three BPNs: (1) *autonomy*, a sense of volition or the need to act freely without external pressure; (2) *competence*, a sense of efficacy or the need to experience oneself as effective and successful; and (3) *relatedness*, the need to feel that one is connected to others, has loving relationships, and belongs to a community.

COVID-19 not only disrupted social norms but also profoundly affected the satisfaction of basic psychological needs (e.g., Calvo et al., 2020; Cantarero et al., 2020) by necessitating social distancing and remote work. Despite this, certain activities, for example, regular physical exercise and routines may have positively contributed to coping with the anxiety induced by the pandemic’s challenges (Bentzen et al., 2021; Brooks et al., 2020; Calvo et al., 2020; Cantarero et al., 2020; Costa et al., 2022; Müller et al., 2021).

Certainly, the COVID-19 pandemic highlighted social relationships’ importance to our physical and mental health (e.g., Bond, 2021; Cooper et al., 2021; Costa et al., 2022; Sommerlad et al., 2022). Specifically, romantic relationships’ critical role was placed under a unique lens (Pietromonaco & Overall, 2021). Challenges imposed by the pandemic—financial insecurities, altered routines, and shared anxieties—tested romantic partners’ resilience (e.g., Candel & Jitaru, 2021; Estlein et al., 2022). While previous research has emphasized relationship quality’s importance for mental well-being (Teo et al., 2013), the connection between the quality of romantic relationships and the fulfillment of basic psychological needs, which serve as protective factors for mental well-being, has remained unexplored in the context of perceived COVID-19 stress, even though people in satisfying relationships reported better mental health during the pandemic. Indeed, *the quality of the relationship* mattered the most for well-being, not just being in a relationship. Thus, a supportive romantic partner might help manage an individual’s emotions related to the COVID-19 situation (e.g., worry or hopelessness) (Randall et al., 2021; Till & Niederkrotenthaler, 2022). Despite evidence that individuals in fulfilling relationships reported better mental health during the pandemic (e.g., Till & Niederkrotenthaler, 2022), no study has simultaneously investigated the interrelationship between basic needs satisfaction, relationship quality, and mental well-being during the COVID-19 crisis and its related stress.

## Present Study

COVID-19-related stress, which encompasses perceptions of threat, concern, and control, has been negatively associated with emotions and well-being (Erbiçer et al., 2021; Pérez-Fuentes et al., 2020; Quigley et al., 2023). This study aimed to provide understanding of the interplay in mental well-being among basic psychological needs, relationship quality, and COVID-19-related stress during the pandemic and quarantine for those living in Hungary. By investigating associations between these variables, our research aimed to provide nuanced insights into mental health’s protective factors and close relationships’ reciprocal nature, examining how COVID-19-related stress impacted both basic psychological need satisfaction and relationship quality.

**Figure 1:**
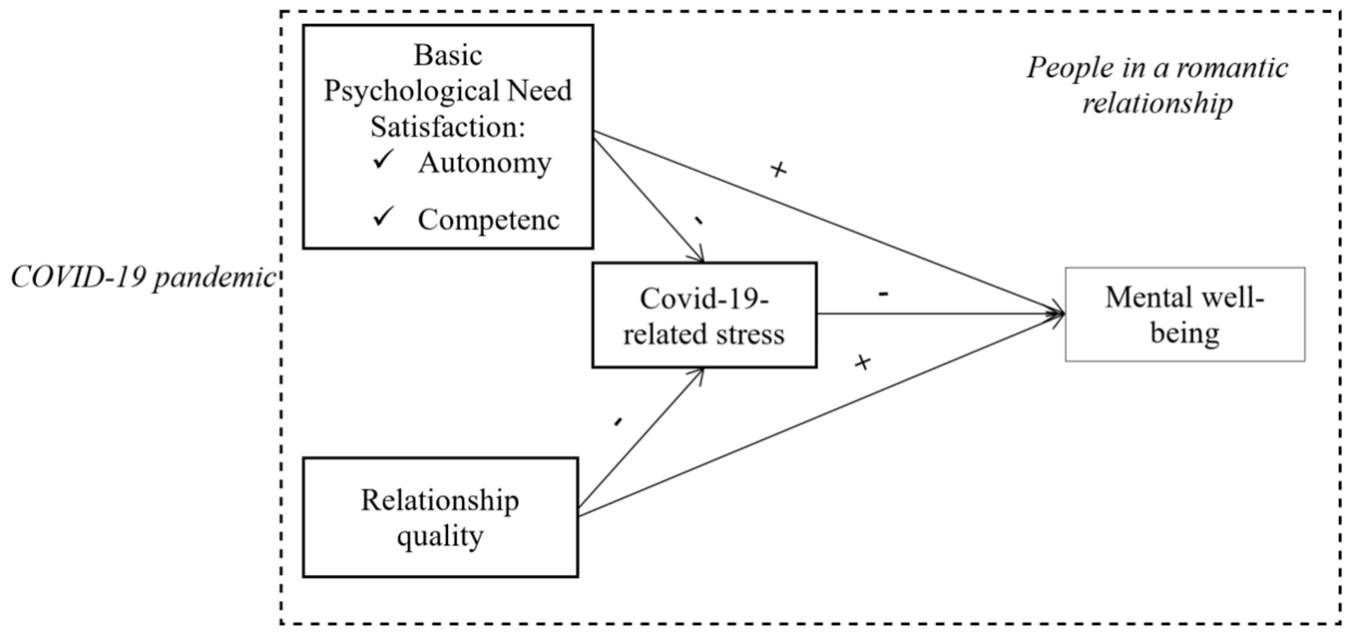
Model Summary With Hypothesized Paths *Note*. “+” stands for positive association, and “−” for reversed association.

Grounded in the basic psychological need microtheory (Ryan & Deci, 2000) of SDT (Ryan & Deci, 2017), we first tested whether BPNs and relationship quality (RQ) predicted lower COVID-19 stress. Next, we investigated whether BPNs and higher RQ positively predicted mental well-being. Finally, we assessed whether COVID-19-related stress predicted lower mental well-being and, thus, partially mediated the predictive power of BPNs and RQ on mental well-being. Figure 1 overviews the model and hypothesized relationships between variables.

Specifically, the following hypotheses (**H**) were tested using structural equation modeling:

**H1:** Basic psychological need satisfaction (i.e., autonomy, relatedness, and competence) are associated with higher mental well-being.
**H2:** Higher relationship quality (RQ) is positively associated with higher mental well-being.
**H3:** Higher COVID-19-related stress predicts lower mental well-being.
**H4:** Higher BPNs predict lower COVID-19-related stress.
**H5:** Higher RQ predicts lower COVID-19-related stress.

## Method

### Recruitment and Participants

This study’s data were collected from March to May 2020 during Hungary’s initial national quarantine. Reached by online request, participant-volunteers were recruited from social networking sites (e.g., Facebook) using convenience sampling.

Participants met the following inclusion criteria: aged above 18 years, in a romantic relationship, and formally consenting to participate. Exclusion criteria were aged under 18 years and currently or within the last year receiving psychiatric treatment. Our 805 participants’ average age was 37.88 ± 12.50 years, and the majority (70.19%) were women. Table 1 summarizes participants’ demographic information.

**Table 1:** Demographic Information

Demographic	*N*	Percent (%)	Mean	*SD*
Age	805		37.88	12.50
Gender				
Female	565	70.19		
Male	240	29.81		
Relationship duration				
Less than 6 months	41	5.09		
More than 6 months, less than 1 year	40	4.97		
More than 1 year	724	89.94		
Living together with partner				
Yes	659	81.86		
No	146	18.14		
Level of education				
High school or less	232	28.82		
BA	268	33.29		
MA and higher	305	37.39		

### Procedures

Taking approximately 20–30 minutes, study participants completed an online survey questionnaire, in which they first received comprehensive information about the research objectives and the terms of their involvement.

### Measures

All procedures performed in the study were following the ethical standards of the institutional, regional and national research committee and with the 1964 Helsinki Declaration and its later amendments or comparable ethical standards. Ethics approval was provided under the Unified Ethics Committee for Psychological Research (EPKEB, Hungary). Throughout the study, we strictly followed ethical guidelines. Documents included in the study (the complete data file, the JASP file including the R code used by JASP, a data code book in Excel format with the variable names and Hungarian and English labels and answer options in English, and the materials used in the study (Ethical approval and the original Limesurvey questionnaire) are available in the “Hungarian Data EJOP Submission 2024” subfolder in the Open Science Framework folder of the main research on Randall et al. (2024).

#### Basic Psychological Needs

Basic psychological needs were measured with the Hungarian version of the Basic Psychological Need Satisfaction Scales (La Guardia et al., 2000; Martos & Szondy, 2023), adapted for everyday life. Reflecting personal experiences with basic psychological need satisfaction, the questionnaire contains nine items on three subscales: *autonomy* (e.g., “I generally feel free to express my ideas and opinions”), *competence* (e.g., “Most days I feel a sense of accomplishment from what I do”), and *relatedness* (e.g., "I really like the people I interact with”). Items were rated on a 7-point Likert-type scale (1 = *Not at all characteristic* to 7 = *Fully characteristic*). The subscales (three items each) demonstrated good internal consistency for *autonomy* (Cronbach’s α = .69), *competence* (Cronbach’s α = .70), and *relatedness* (Cronbach’s α = .70).

#### Relationship Quality

Relationship quality was measured with the Perceived Relationship Quality Components Inventory (Fletcher et al., 2000; Martos & Sallay, 2023b, designed to measure relationship satisfaction. The scale consists of six subscales with three items each: *relationship satisfaction* (e.g., “How satisfied are you with your relationship?”), *commitment* (“How committed are you to your relationship?”), *intimacy* (“How intimate is your relationship?”), *love* (“How much do you love your partner?”), *passion* (“How passionate is your relationship?”), and *trust* (“How much do you trust your partner?”). Items are rated on a 3-point response scale (1 = *Not at all*, 2 = *Neutral*, 3 = *Strongly*). We used the total average score, and the questionnaire demonstrated good internal consistency (Cronbach’s α = .94; 18 items).

#### Perception of COVID-19-Related Stress

The level of COVID-19-related stress was measured with the Hungarian version of the 7-item Brief Illness Perception Questionnaire (Broadbent et al., 2006; Látos et al., 2021). In this study, we used four stress-related items from the questionnaire, modified for the COVID-19 context (e.g., “How concerned are you about the COVID-19 outbreak?” and “How well do you feel you understand the situation with COVID-19?”). Items were rated using a 10-point Likert-type scale (0 = *I am not at all concerned* to 10 = *I am very concerned)*. Even though the BIPQ-7 subscale’s internal consistency was less than ideal (Cronbach’s α = .58; four items), we decided to retain the score for further analysis.

#### Mental Well-Being

To measure well-being, we used the Hungarian version of the Warwick–Edinburgh Mental Well-Being Scale (WEMWBS; Martos & Sallay, 2023a; Tennant et al., 2007), which contains 14 items on positive emotions, satisfying interpersonal relationships, and positive functioning (e.g., “I’ve been feeling optimistic about the future”; “I’ve had energy to spare”). Items were rated on a 5-point Likert-type scale (1 = *None of the time* to 5 = *All of the time*). The total scale score was calculated by summing the 14 individual item scores. Higher scores indicate higher well-being. The questionnaire exhibited good internal consistency (Cronbach’s α = .91; 14 items).

## Results

### Correlational Analyses

JASP 0.14.6.0 was used for the data’s statistical analysis. Table 2 shows the weak, significant negative correlation between the total score for mental well-being and COVID-19-related stress: *r_p_* (805) = −.30, *p* < .001. Our results indicate a weak to medium-strong, positive, significant correlation among autonomy, competence, relatedness satisfaction, and mental well-being. Specifically, autonomy and mental well-being showed a medium, significant positive correlation: *r_p_* (805) = .50, *p* < .001; competence and mental well-being showed a medium, significant positive correlation: (805) = .52, *p* < .001; and relatedness and mental well-being showed a weak, significant positive correlation: *r_p_* (805) = .35, *p* < .001. Finally, we examined the correlation between relationship quality and mental well-being: *r_p_* (802) = .28, *p* < .001.

**Table 2:** Correlation Matrix and Participants’ Characteristics and Psychological Variables

Variable	*M*	*SD*	1	2	3	4	5	6
1. BPNS Autonomy	4.81	1.29	1					
2. BPNS Competence	4.58	1.39	.548***	1				
3. BPNS Relatedness	5.87	1.00	.513***	.363***	1			
4. PRQC Total Score	2.69	.38	.225***	.153***	.209***	1		
5. Covid-19-related stress	5.00	1.32	-.305***	-.179***	-.028	.062	1	
6. WEMWBS Total Score	3.63	.62	.499***	.515***	.345***	.281***	-.302***	1

*Note.* Minimum-maximum values: PRQC: 1–3, BPNS: 1–7, WEMWBS: 1–5, BIPQ: 1–10.

*p < .05. **p < .01. ***p < .001. denote significant values.

### Path Analysis

**Figure 2:**
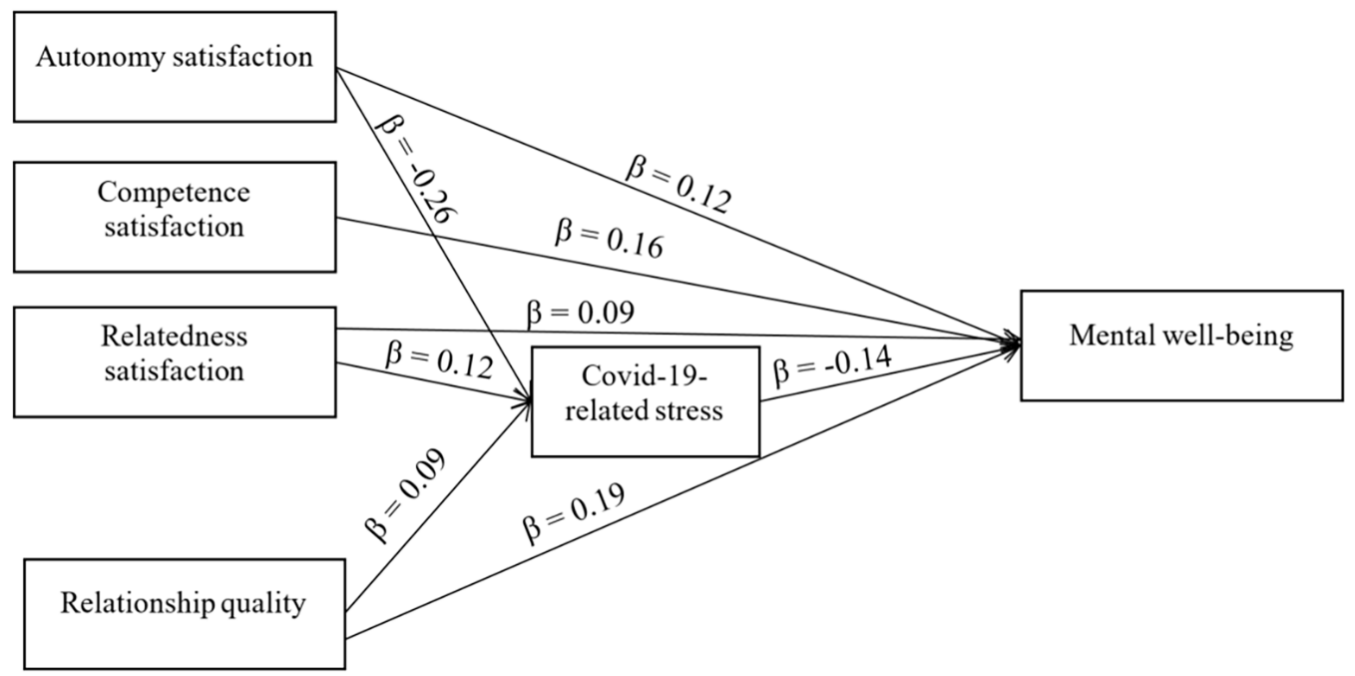
Path Model *Note.* Only significant paths are included.

Structural equation modeling was used to investigate the associations in a multivariate manner. Model fit was tested using the following indices (Hu & Bentler, 1999): Chi-square statistic (χ^2^), comparative fit index (CFI), normed fix index, Tucker Lewis Index or Nonnormed Fit Index (TLI or NNFI), root mean square error of approximation (RMSEA), and standardized root mean square error of approximation. These indices indicated good fit to the data: χ^2^ (10) = 56.64, *p* < .001, CFI = .96, TLI = .81, RMSEA = .08, SRMR = .034. Table 3 summarizes the model’s defined paths and standardized parameter estimates of direct effects. The variables explained 41% of WEMWBS variance (*R*^2^ = 0.41). See Figure 2 for depiction of significant paths.

**Table 3:** Standardized Parameter Estimates of Direct Effects for the Model

Parameter	Estimate	*z*	*p*
Autonomy → COVID-19 stress	-0.26	-5.82	< 0.001
Competence → COVID-19 stress	-0.01	-0.33	0.74
Relatedness → COVID-19 stress	0.12	2.98	0.003
RQ → COVID-19 stress	0.09	2.63	0.008
Autonomy → WEMWBS	0.12	6.08	< 0.001
Competence → WEMWBS	0.16	8.97	< 0.001
Relatedness → WEMWBS	0.09	2.61	0.007
RQ → WEMWBS	0.19	6.95	< 0.001
COVID-19- stress → WEMWBS	-0.14	-4.88	< 0.001

*Note*. In the model beyond the variables presented in the table, associations were controlled for gender, age, level of education and relationship duration.

Acronyms: RQ = Relationship Quality, WEMWBS = Warwick–Edinburgh Mental Well-Being Scale.

### Hypotheses 1, 2, and 3: Predictors of Mental Well-Being

According to structural equation modeling, mental well-being was significantly and positively predicted by autonomy (β = .12, *p* < .001), competence (β = .16, *p* < .001), relatedness (β = .09, *p* = .007), and relationship quality (β = .19, *p* < .001). It was significantly and negatively predicted by COVID-19-related stress (β = -.14, *p* < .001).

### Hypotheses 4 and 5: Predictors of COVID-19-Related Stress

COVID-19-related stress was significantly negatively predicted by autonomy (β = -.26, *p* < .001) and significantly positively predicted by relatedness (β = .12, *p* = .003) and relationship quality (β = .09, *p* = .008). COVID-19-related stress was not significantly predicted by competence (β = -0.01, *p* = .74).

## Discussion

This study investigated the COVID-19 pandemic’s psychological impact, focusing specifically on satisfaction of basic psychological needs (autonomy, competence, and relatedness), relationship quality in romantic relationships, and perceived COVID-19-related stress. Hypothesis 1 on the relationship between basic psychological needs (BPN) satisfaction and mental well-being indicated that, indeed, individuals experiencing autonomy, competence, and relatedness during quarantine exhibited better mental health. This aligns with prior research indicating that satisfying basic psychological needs can act as a protective factor for mental health in challenging circumstances, such as the COVID-19 pandemic. Consistent with the principles of Self-Determination Theory (SDT) proposed by Ryan and Deci (2017), activities that fulfill autonomy, competence, and relatedness needs contribute to well-being and alleviate stress in challenging situations (Behzadnia & FatahModares, 2020; Weinstein et al., 2016). The ability to choose activities autonomously and engage in self-regulated behavior leads to increased vitality, reduced stress, and enhanced well-being, even during social distancing or lockdowns (Behzadnia & FatahModares, 2020). Learning activities considered personally significant and taking responsibility for oneself have also proven beneficial (Ryan & Deci, 2017; Weinstein et al., 2016). These findings have important implications for healthcare professionals, the need to develop mental health preventive programs that address basic psychological needs.

The second hypothesis on the association between mental well-being and relationship quality, revealed that a higher RQ predicted better mental well-being. This is consistent with existing research according to which that emotionally satisfying romantic relationships and positive relationship processes can buffer stress in general (Randall & Messerschmitt-Coen, 2019) and specifically in crises (Randall et al., 2021).

The third hypothesis, focused on the association between COVID-19-related stress and mental well-being, was confirmed, with a higher level of perceived stress predicting lower mental health. This aligns with prior studies, indicating that a more threatening perception of the COVID-19 pandemic is associated with increased feelings of irritation and lower well-being (Erbiçer et al., 2021; Pérez-Fuentes et al., 2020; Quigley et al., 2023).

The fourth and fifth hypotheses identified predictors of COVID-19-related stress. Analysis partially supported Hypothesis 4; only autonomy significantly and positively predicted COVID-19 stress. In contrast, competence did not significantly predict perceived stress, whereas relatedness satisfaction predicted COVID-19 stress in the opposite direction. When considered with the results of Hypotheses 2 and 5, these associations suggest that in globally stressful situations like the COVID-19 lockdown, relationships may not only serve as protective factors but also as sources of vulnerability. The positive association with well-being confirms previous research findings that better relationship functioning can buffer COVID-19-related mental problems (e.g., Randall et al., 2021). However, satisfying relationships may not only contribute to well-being but also make the individual perceive the stressful situation as more threatening. Experiencing a loving and reciprocal relationship in a stressful situation where everybody is affected and uncertainty prevails, COVID-19 might have been perceived as more threatening due to concerns for close others. (Fino et al., 2022; Heanoy et al., 2021; Sams et al., 2023; Whitehead & Torossian, 2021).

### Strengths and Limitations

Although these observed associations may be particularly relevant to the pandemic’s early phase, they also offer meaningful insights into the crucial role of basic psychological needs and relationships in mental health during times of uncertainty and stress. However, we must acknowledge certain limitations in our study. Reliance on online data collection, necessitated by lockdown restrictions preventing in-person surveys, is a noteworthy constraint. Despite the sizable number of respondents, the sample cannot be considered fully representative, given the inherent biases associated with convenience sampling. This methodological choice resulted in an uneven distribution across gender, age, and education levels. In addition, the research’s cross-sectional design imposes constraints on the results’ interpretative scope, offering only a snapshot rather than dynamic understanding of the studied relationships. Another limitation arises from initial use of certain instruments on a Hungarian sample, potentially introducing bias. Adoption of validated questionnaires would have enhanced the results’ comparability and reliability. Notably, the BIPQ-7 questionnaire exhibited low internal consistency, further highlighting a limitation in our measurements’ precision. Finally, although significant, many associations were weak, suggesting that the studied phenomena cannot be simplified to individual factors and that inclusion of multiple measures may provide a more comprehensive model.

### Conclusion

This study investigated the impact of the COVID-19 pandemic on mental health, specifically focusing on the satisfaction of basic psychological needs (autonomy, competence, and relatedness), relationship quality in romantic relationships, and perceived COVID-19-related stress. Despite its limitations, the study’s novel contributions and insights remain valuable for advancing understanding of the complex dynamics between basic psychological needs satisfaction, relationship quality, stress, and mental well-being in the context of a global crisis such as the COVID-19 pandemic. Importantly, among the basic psychological needs, satisfaction of the need for autonomy directly and indirectly predicted higher well-being by reducing perceived stress, highlighting the crucial role autonomy might play in global situations of uncertainty and constraints.

### Clinical Implications

Despite intricate challenges posed by the pandemic and other global uncertainties, our aim was to provide profound insights into mental health protection factors and the social environment’s impact during times of adversity. Understanding factors that shape individuals’ mental well-being in crises such as the COVID-19 pandemic is of paramount importance. This study has considerable implications for shaping interventions and support systems to address specific psychological needs and relationship dynamics during crises. By integrating our findings into mental health interventions, we anticipate heightened efficacy in aiding individuals as they navigate challenging situations, ultimately contributing to improved overall mental health outcomes.

## Supplementary Materials

For this article, the following Supplementary Materials are available:

- Data. (Randall et al., 2024)
- Code. (Randall et al., 2024)
- Study materials. (Randall et al., 2024)
- Preregistration. (Randall et al., 2020)

## Data Availability

For this article, data, code and study materials are available at Randall et al. (2024). The preregistration is available at Randall et al. (2020)
